# The impact of COVID-19 on PRO development, collection and implementation: views of UK and Ireland professionals

**DOI:** 10.1186/s41687-023-00663-y

**Published:** 2023-11-27

**Authors:** Patricia Holch, Grace Turner, Anju D Keetharuth, E Gibbons, Kim Cocks, Kate L Absolom

**Affiliations:** 1 Department of Psychology, School of Humanities and Social Sciences, Leeds Beckett University, Portland Building, Room PD402, City Campus, Leeds, LS1 9HE UK; 2 Institute of Applied Health Research, College of Medical and Dental Sciences, University of Birmingham, Edgbaston, Birmingham, B15 2TT UK; 3 School of Health and Related Research, Regent Court, 30 Regent Street, Sheffield, S1 4DA UK; 4 Evidera Ltd, 201 Talgarth Rd, The Ark, London, W6 8BJ UK; 5 Adelphi Values, Patient-Centered Outcomes, Bollington, Cheshire, SK10 5JB UK; 6 Leeds Institute of Medical Research, University of Leeds, St James’s Hospital, Bexley Wing, Beckett Street, Leeds, LS9 7TF UK; 7 Leeds Institute of Health Sciences, University of Leeds, Leeds, LS2 9JT UK

## Abstract

**Background:**

PROs are valuable tools in clinical care to capture patients’ perspectives of their health, symptoms and quality of life. However the COVID-19 pandemic has had profound impacts on all aspects of life, in particular healthcare and research. This study explores the views of UK and Irish health professionals, third sector and pharmaceutical industry representatives and academic researchers on the impact of COVID-19 on PRO collection, use and development in clinical practice.

**Methods:**

A volunteer sample took part in a 10 question cross sectional qualitative survey, on the impact of COVID-19, administered online via Qualtrics. Demographic data was descriptively analysed, and the qualitative free text response data was subject to thematic analysis and summarised within the Strengths, Weaknesses, Opportunities and Threats (SWOT) framework.

**Results:**

Forty nine participants took part located in a range of UK settings and professions. Participants highlighted staff *strengths* during the pandemic including colleagues’ flexibility and ability to work collaboratively and the adoption of novel communication tools. *Weaknesses* were a lack of staff capacity to continue or start PRO projects and insufficient digital infrastructure to continue studies online. *Opportunities* included the added interest in PROs as useful outcomes, the value of electronic PROs for staff and patients particularly in relation to integration into systems and the electronic patient records. However, these opportunities came with an understanding that digital exclusion may be an issue for patient groups. *Threats* identified included that the majority of PRO research was stopped or delayed and funding streams were cut.

**Conclusions:**

Although most PRO research was on hold during the pandemic, the consensus from participants was that PROs as meaningful outcomes were valued more than ever. From the opportunities afforded by the pandemic the development of electronic PROs and their integration into electronic patient record systems and clinical practice could be a lasting legacy from the COVID-19 pandemic.

## Introduction

Patient Reported Outcomes (PROs) are valuable tools for clinical care and research to capture patients’ perspectives of their health, symptoms, decision making and quality of life [1–6]. The COVID-19 pandemic has had profound impacts on virtually all aspects of life, including healthcare and research.

In addition to the direct burden of treating COVID-19, clinical care has further been affected by redeployment of staff, reorganisation of healthcare services, delayed or rescheduled non-essential medical activities, and staff sickness and burnout [7–9]. From a research perspective, millions of pounds has been invested in funding COVID-19 research; however, there has been a detrimental impact on non-COVID research, including study closures, limited funding and reduced capacity of researchers due to illness and caring commitments [10–12]. Together, these unique challenges have had a significant impact on the development and collection of PROs. However, there is also some evidence of COVID-19 creating positive opportunities and innovations, such as advances in the adoption and implementation of electronic PROs in both clinical trials and practice [13, 14].

The aim of this study was to explore the views of a wide range of stakeholders who are involved with PRO development and implementation in the UK. Therefore health professionals, third sector (including charities, voluntary and community group) representatives, pharmaceutical industry representatives and academic researchers were asked about the impact of COVID-19 on PRO collection, use and development in clinical practice and research in the United Kingdom (UK) & Ireland. The findings will be explored using the strengths, weaknesses, opportunities and threats (SWOT) framework [15]. The Strengths Weaknesses Opportunities and Threats (SWOT) framework is a strategic analysis tool that enables exploration of the challenges between external factors (opportunities or threats) and internal capacities and competencies (the strong and weak points of an organisation) to enable strategic planning. While its use is most common in private companies [16] it is also adopted more broadly by government organisations including health care [17].

This seemed an ideal framework within which to view the threats posed and opportunities afforded by the COVID-19 pandemic on PRO development, integration and collection, whilst recognising the inherent external and internal weaknesses and strengths.

## Method

### Design

A cross sectional survey with free text comments was utilised. Given the time and resource constraint and the nature of the questions, the survey methodology was deemed appropriate to enable the exploration of participants’ views at a single time point [15] between November 2020 and April 2021 (covering the UK COVID-19 first and second national lockdowns. Participants were recruited as a volunteer sample, via i) the ISOQOL UK & Ireland special interest group communication forum (Teamwork) and ii) email lists from delegates (who agreed to email contact) from the 2019 UK PROMS conference. The methodology is modelled on similar surveys conducted with British Psychosocial Oncology Society members [12, 18].

### Materials

**Fig. 1:**
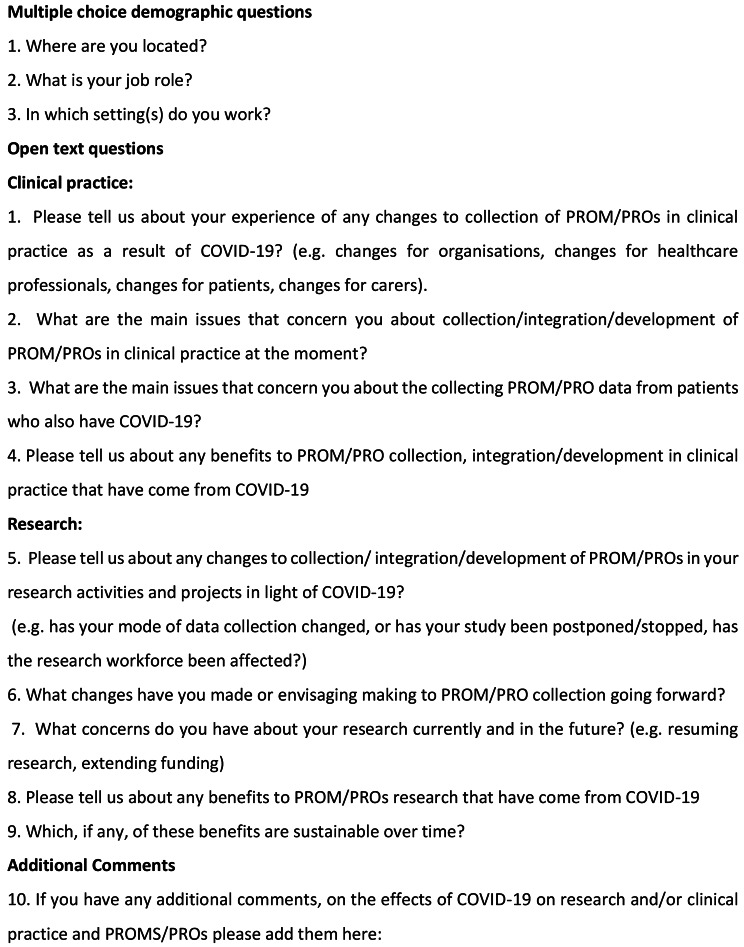
Survey questions

The survey was administered via an online questionnaire builder Qualtrics ™ between November 2020 and April 2021 (covering the UK COVID-19 first and second national lockdowns). Participants completed three brief demographic questions about their role, area, location followed by 10 questions focussed on the impact of COVID-19 (Fig. 1). Researchers were asked about any changes to collection/ integration/development of PROs in their research activities and projects as a result of COVID-19; changes they have made or were envisaging making to PRO collection in the future; concerns about their research at the time and in the future; any benefits to PRO research that came from COVID-19 and whether these benefits were sustainable over time. Those involved in clinical practice were asked questions around the main issues that concerned them about collection/integration/development of PROs in clinical practice at the time; the main issues that concerned them about collecting PRO data from patients who also had COVID-19; the benefits to PRO collection, integration/development in clinical practice that arose from COVID-19 (Fig. 1).

### Participants

We aimed to recruit UK and Ireland based professionals working in the field of PROs in the UK and Ireland including clinicians, academics, allied health professionals, nurses and those working in pharmaceutical industry and the third sector.

### Procedure

An invitation to complete the online survey was circulated via the International Society for Quality of life UK and Ireland Special Interest Groups mailing list, previous PROMs UK conference mailing lists and via social media platforms. The information sheet advised on the content of the survey, participants right to withdraw and how their data would be used and stored.

### Analysis

All demographic data was descriptively analysed using IBM SPSS version 26. The qualitative free text response data was subject to the six stages of thematic analysis; familiarisation, generating initial codes, searching for themes, reviewing themes, defining themes and reporting the findings [19, 20]. We initially employed a deductive approach to coding using the SWOT framework (used for health service development and reform) as main theme headings and further inductive coding explored sub themes under the SWOT framework. The research team coded several questions each and these were second coded by the lead author Patricia Holch (PH).

## Results

Forty nine participants completed the survey (see Table 1) located in a range of settings and professions; academic researcher, n = 24 (32%), doctor, n = 9 (12%), pharmaceutical industry representatives = 6 (8.0%), the majority of participants n = 18 (24%) were from the Yorkshire and Humber region.

**Table 1:** Sociodemographic details of the sample (N = 49*)*

Geographical location	N (%)	Job role	N (%)
Yorkshire & Humberside	18 (24%)	Academic researcher	24 (32%)
London	7 (9.3%)	Doctor	9 (12.0%)
South East	5 (6.7%)	Industry	6 (8.0%)
South West	4 (5.3%)	Allied Health Professional	3 (2.7%)
North West	4 (5.3%)	Psychologist	2 (2.7%)
Wales	4 (5.3%)	Health Service Manager	1 (1.3%)
Northern Ireland	2 (2.7%)	Clinical Academic Researcher	1 (1.3%)
Scotland	1 (1.3%)	Charity	1 (1.3%)
		*Missing	2 (2.7%)
**Total**	49 (100%)	**Total** 47 (95.9%)	

*This refers to missing data

Below is a summary of themes identified from the qualitative survey organised under the SWOT framework supportive quotes can be viewed underneath the narrative.

### Strengths

#### Embracing new modes of communication

Participants discussed how by necessity, they adapted and embraced new remote ways of communication (e.g. videoconferencing) which proved cost effective, flexible and accessible for patients who could not come in to hospital, explained by this academic researcher:

*“Increased familiarity with videoconferencing has had benefits to arranging e.g. PPI meetings to inform PRO research - making meetings easier to organise, cheaper and more accessible to participants*” [AR,16].

Embracing new ways of communication has facilitated research related work and enhanced communication between colleagues and patients, here illustrated by an industry academic:

*“Increasing our ability to engage with patients through telemedicine…”* [IND, 1].

Further, this academic researcher described how this how these collaborative ways of working was viewed effective and valuable to staff:

*“Acceleration of work as people work more collaboratively***”….***is a definite advantage, I think people now see the necessity of working together, more than ever before. I hope the ‘team’ ethos will continue to reap benefits for all”* [AR, 18].

#### Staff flexibility

During the pandemic staff have had to be flexible to enable the continuation of research projects with quick changes required from research teams to continue with PRO research. Participants expressed concerns about conventional (paper) data collection, highlighting the need to consider additional reminders, and if the latter did not translate into responses, they would engage with public and patient involvement (PPI) to co-design engagement strategies. Others have resorted to more telephone and postal modes of administering study materials, illustrated here:

“*Our procedures for recruitment and follow up will now be mainly online/phone. This is all very new, and we are trying to ensure we are doing everything right, but we really don’t know how it will all work in practice”* [AR,14].

#### Existing embedded electronic PRO (ePRO) collection

The NHS (National Health Service) hospitals that already had existing ePROs embedded within their systems, according to this academic researcher were most likely to buffer the challenge of COVID-19 on PRO collection:

*“All data collection was electronic - so no issues with mode of administration*” [AR, 9].

As these hospitals were already set up for remote data collection, the changes the majority of hospitals had to implement were not required and thus data collection could continue as normal to the benefit of research and clinical practice. Thus, full electronic integration was considered seen as a desirable goal for trusts in the future:

*“Movement solely to electronic data collection and not using any paper forms*” [AHP, 1].

### Weaknesses

#### Staff capacity

The pandemic has resulted in staff being deployed elsewhere, and adapting to new working processes, thus the capacity of staff for PRO development or collection was limited described by this doctor: *“There is little capacity for doing so. Focus is on challenges of capacity and new ways of working*…workforce redeployed to clinical duties research studies abandoned” [DR,2].

There was a lack of staff capacity for PRO research and minimal financial resources to continue PRO research described by this hospital consultant:

“*It is incredibly difficult as it is not given any time or resource; as such it is essentially an extracurricular activity for the enthusiasts*” **[DRC,1]**.

#### Maintenance of electronic infrastructure

COVID-19 has exposed the lack of stable infrastructure within organizations for remote digital data collection, and the lack of co-ordination within and between organisations to facilitate large scale uptake:

*Patient-facing digital solutions could be really beneficial to collecting PROs but at present there doesn’t appear to be much coordination around this and each service or Trust is seeking their own solutions”* [CG, 1].

Participants also highlighted that even if the digital infrastructure were available, there would be a need for staff and patients to be trained to use these systems for them to be optimised to full capacity, Participants also highlighted that even if the digital infrastructure were available, there would be a need for staff and patients to be trained to use these systems for them to be optimised to full capacity:

*“Initiatives or services previously implemented have had problems: maintaining the systems up-to-date (due to staff being poorly or working remotely), electronic systems not being appropriately maintained on a constant basis; new staff starting in roles using such systems have had issues accessing, using them and hence gaining the necessary experience”* [AR, 17].

#### Digital exclusion

In health systems with embedded electronic infrastructure, electronic and telemedicine approaches were quickly embraced during the pandemic. However, these academic researchers highlighted that not all patients can embrace electronic collection of PROs, particularly the elderly or those of low economic status and that data quality may be impaired due to remote completion:

“*Not everyone has online access…whether patients have online access to fill them in…*” [AR, 19].

*“Not all patient groups I work with do well with online data collection. Not being able to assist completion of paper-based questionnaires may well affect the amount and quality of data collected”* [AR,4].

*“A lot of positive changes in terms of remote data collection but risk of discriminating against people who can’t access remote communication*” [AR, 6].

#### Lack of PRO integration in electronic patient record (EPR) systems

The pandemic instigated the requirement for quick changes in modes of data collection (from face-to-face to electronic) to enable the continuation of PRO studies/clinical implementation. However, it became clear that many hospitals would be faced with operational challenges because of the lack of PRO integration into their EPR systems where staff could review the results:

*“Not everyone has online access…whether patients have online access to fill them in and then whether they are accessible with the hospital online results system*” [AR, 19].

*“…. Challenges integrating PROM data into clinical record…*” [AR,22].

“*Access to electronic questionnaires needs improvement*…” [IA 4].

#### Lack of COVID-19 PROs

Doctors voiced concerns about the absence of validated PROs to collect meaningful data given the new symptoms patients were presenting with, due to COVID-19:

*“It’s difficult to find validated instruments to capture the impact of the pandemic on quality of life*”[DR, RF, 2].

A psychologist also suggested that COVID-19 could disrupt the validity of the results from existing disease specific PROs due to emerging symptoms that were not included in these measures.

“*I would be concerned about the impact of (COVID) physical symptoms influencing the responses*”. [PSYC,3]

### Opportunities

#### Electronic collection of PROMs/PROs

A clinical academic identified that one of the major opportunities afforded by the pandemic was the willingness of both staff and patients to embrace online (electronic collection of PROs):

*“Many appointments are now down by phone so there needs to be remote ways of collecting PRO data*” [CA, 1].

Furthermore, doctors stated the risk of COVID-19 resulted in patients being more willing to accept online completions of PROs and with more staff appreciating the value of electronic capture of PROs for research and clinical practice:

*“However, with remote working my clinical colleagues realised the value of having symptom data collected via PROMs and approached me to help build a business case for several cancer sites*” [DRC, 5].

“we are currently using the opportunity to establish an online tool through Qualtrics” [DRRF,1] Participants also highlight that the adoption of electronic data capture in health systems could be a lasting legacy of COVID-19:

*“The use of online remote reporting of PROMS/PROs may be one of the things which benefit, in terms of utilisation and popularity, from the pandemic in the longer term*” [AR,5].

“*Remote review is the future*” [DR,3].

#### Upscaling of electronic PRO systems

A number of participants including doctors and academic researchers described that there was a renewed focus within the health systems to continue and expand the use of electronic PRO systems:

“*We have renewed focus on transitioning to electronic collection of proms on an organisational wide leve*l” [DR,CF1].

“…*I hope that the rapid scaling up of electronic data capture and consultation systems within the NHS will result in a system of infrastructure within which ongoing collection of PRO data can be more easily realised*” [AR,7].

#### Remote clinical consultations

Both clinical academics and health service mangers stressed that the advantages afforded by remote data collection during the pandemic could have positive implications for the future of clinical practice, offering significant advantages and opportunities rather than seen as an inferior solution. In addition, these consultations could include PRO collection which ideally could be integrated into the EPR systems.

*“Creating a realisation that online and remote consultations are a valuable part of a clinical service rather than only second best*” [CA, 1].

*“Preparation of a business case for a new electronic health record - release of funds Video outpatient appointments, incorporating use of PROMs*” [HSM, 1].

An industry academic indicated that moving forward clinical assessments in clinical trials could also be transformed by the use of electronic technology:

“*Looking into biosensors and telemonitoring to support clinical trials instead of clinical assessments during study visits*” [IA, 1].

#### Value of PROs

Consultants and health service managers stressed how the pandemic highlighted the value of collecting patient reported data via PROs to clinicians and patients, particularly for long COVID symptoms. Staff have realized the importance of capturing short and long-term outcomes that are meaningful to patients and carers.

.*”increased focus on outcomes and collection of data, new focus on outcomes related to carers and communication tools - the nonclinical aspects of care”* [DR, C2].

*“ Increased understanding that using mortality as the main outcome measure is not good enough – long Covid has made us think about longer term outcomes that matter to patients*” [HSM, 1].

*“In my opinion a failure to measure the end result of care hinders improvement at a patient and population level*” [DRC, 1].

### Threats

#### Access to patients

One of the major threats identified by participants from COVID-19 was the reduced time and opportunity for PRO collection. This was mainly due to the restrictions limiting the number of patients attending hospital, there were also and fewer staff available within clinical areas for research due to remote working, or being deployed elsewhere.

*“Reduced numbers of patients has reduced the amount of data collection*” [AHP, 1].

In addition, patients were understandably feeling anxious about leaving their homes for appointments and being involved in studies outside their immediate clinical needs, there was less face to face time to collect PROs:

*“ Less face to face time to collect PROs. Completing PROs can be seen as taking up time and patients may not want to be in clinic longer than necessary for their treatment…* t*his will affect PRO collection and indicate a push towards developing reliable and accessible remote methods”.*” [CA, 1].

#### Development projects on hold and research stopped /delayed

Numerous PRO collection and development projects were either stopped or paused due to the reduced time and opportunity. This was particularly true for areas where face-to-face paper PRO collection was employed:

*“Prior to Covid we were collecting outcome measures in paper from in-session at assessment at discharge. Since Covid we have not been able to collect any outcome measure data….***”** [PSYC, 3].

*“essentially all PROMs stopped due to emphasis on arranging appointments and managing patients remotely. This was prioritised and we have no ability to collect data systematically.*” [DRC,2].

Often studies were indefinitely on hold with staff forgoing the opportunity of collecting longitudinal data.

“*Loss of data at pre-determined time points after a specific clinical intervention. Lack of engagement of participants. Further research restrictions during the current peak*” [DRRF,1].

For studies to continue, where possible, research staff had to redesign studies to be conducted online:

*“The main result of this has been that primary data collection is either indefinitely on hold….or is having to be redesigned to prioritise remote data collection (e.g. starting data collection through an online survey rather than individual interviews)”* [AR,7].

#### Low priority of PROs

There was a need for services to prioritise treating COVID-19 within hospitals and academic institutions and university ethics committees prioritised COVID-19-related research. s highlight concerns that the development and implementation and collection of PROs would not be prioritised due to the necessity of clinical staff and managers to focus on COVID-19.

“*As a researcher trying to support the integration of PROMs into clinical practice, my main concern is that this has become low priority for clinicians in the context of COVID-19, so it might be harder to engage with them about taking PROMs implementation work forward”* [AR,7].

“*Concern would be whether in the current climate QOL PROs are regarded as a priority”* [AR,19].

*“The main issue to me is that it’s seen by most in management as a ‘nice to have’ rather than a ‘must have’. In turn people prioritise other issues over proms that they view subjectively as more important It then gets pushed down the agenda”***[**DRC, 1].

### Funding disruption

Research funding opportunities during COVID-19 were either suspended or restricted and this has impacted PRO development and integration studies. There were also concerns from participants that funding streams would take time to recover or not fully recover in the post pandemic period.

*“Our major concern is that some funders have suspended funding therefore in future we may not be able to access funding for more research…”* [AR,12].

## Discussion

A qualitative approach was undertaken to understand the impact of COVID-19 on PRO researchers, clinicians, academics, pharmaceutical industry representatives and third sector members in the UK and Ireland. We have structured the findings using the SWOT framework [15] to fully understand the strengths, weaknesses, opportunities and threats posed to PRO development, collection and implementation in the context of COVID-19. The findings highlighted how the impact of COVID-19 caused huge disruption as well as providing opportunities for the future of PRO development, integration and collection in the UK and Ireland.

Strengths included the resilience and agility of staff working within the PRO field to adapt quickly to new ways of working and to embrace novel communication methods. Further, participants described colleagues working more collaboratively within their organisations. These findings concur with research recognising the agility of staff [21] and the value of the human workforce during the pandemic [22]. The value of public and patient involvement (PPI) engagement was evident as one of the most important aspects to the development and integration of PROs. Staff consulted their patient experience groups to support their projects during methodological changes, indeed previous research has shown that PPI groups are integral to the development and delivery of PROs [23, 24]. Together, these challenges born out of the response to COVID-19 have clearly enhanced the research environment in health care settings and beyond.

The weaknesses identified were that organisations were at very different stages of preparedness for online data collection. Those that already had access to ePROs and had ePROs embedded into their EPR systems, could adapt more quickly to the challenges of COVID-19 by transferring all data collection online. However, others were still in the planning stages and did not have the infrastructure available. In general, participants were concerned about both their present and future research. The lack of PROs available to measure the impact of COVID-19 and the impact of the disease on existing data collection was highlighted. Certainly, the medical community in general recommended their prioritisation particularly to measure symptoms and help the treatment of long COVID [25]. Indeed, the first studies relating to PROs to measure the effects COVID-19 are now being published [26–28] including important work from the International Consortium for Health Outcomes Measurement (ICHOM) COVID-19 Working Group [29].

The impact of COVID-19 caused huge threats to PRO development, attributed to delays mainly in ethical and governance approvals and a reluctance of NHS sites to embark on new projects. Threats to PRO collection included the lack of opportunity where ePROs were not available, appointments being conducted remotely, difficulty in engaging participants in research over the telephone compared with face-to-face and researchers themselves being away ill [30]. The delay and interruption to research during COVID-19 was particularly hard for PhD candidates and early career researchers, who are described in the literature as a ‘lost generation’ [31, 32].

One of the major opportunities identified was that respondents are already making or planning to make more widespread use of online modes of data collection. However ePRO application will not be without challenges which include the lack of available resources to train staff and to support patients, the absence of electronic versions of PROs given that even newly developed PROs are sometimes only available for pen and paper administration. Further, whilst there is support in the literature for equivalence across modes of administration (paper and pen vs. ePRO) [33], this is an even more pertinent issue now and warrants further attention among researchers. There have been challenges of approaching participants and obtaining informed consent online. There was also the recognition that online data collection is not appropriate for all patients and that there will be inevitable inequity caused by those unable to complete data online. Although there were some signs that less advantaged populations adapted to digital health care during COVID-19 [34], in the main the digital divide was a threat to equitable care particularly for the elderly and disadvantaged [35, 36]. It is clear however that capturing the patient voice electronically is here to stay and as such, narrowing the digital divide in health care needs to be a priority. There is also a clear desire from respondents to integrate online data collection within clinical practice and trials and existing EPR systems. There is guidance literature on how to successfully integrate PROs within clinical practice which can support this process [37, 38]. However, to integrate PROs fully into clinical care and into EPR systems will require a more widespread and co-ordinated commitment from multiple agencies and stakeholders with dedicated financial resources before this could be realised [39, 40].

This is the only survey to explore the impact of COVID-19 on PRO development, collection and implementation from PRO researchers, clinicians, academics, pharmaceutical industry representatives and third sector members in the UK and Ireland. This has resulted in an in-depth exploration of their views, thus we have gained an understanding of the challenges COVID-19 posed and conversely the opportunities for advancing the field arising from the pandemic. However, as recruitment and data collection occurred during the pandemic, not all potential responders answered the survey. In terms of geographical location participants were primarily located in Yorkshire, Humber and London with other UK regions less well represented. This may have had an impact on the generalisability of these findings to the whole of the UK and Ireland. Similarly, charities and nurses were poorly represented in the survey but the survey did achieve an equal representation from academics and clinicians and their views have offered important insights on PRO development, integration and collection during these unprecedented times.

## Conclusion

Although most patient reported outcome research was temporarily suspended during the pandemic, the consensus from participants was that patient reported outcomes were valued more than ever. From the opportunities afforded during this time, the development of electronic patient reported outcomes and their integration into electronic patient records systems and clinical practice could be a lasting legacy from the COVID-19 pandemic.

## Data Availability

The datasets used and/or analysed during the current study are available from the corresponding author on reasonable request.

## Abbreviations

AR: Academic researcher
AHP: Applied Health Professional CA:Clinical academic
CG: Clinical Governance
DR: Doctor
DRC: Doctor Consultant
DRRF: Doctor Research fellow
HSM: Health Service Manager
IA: Industry Academic
PH: Patricia Holch
PSYC: Psychologist
NB: Numbers, refer to the participant identification numbers in each professional category
